# Long‐Term Population Dynamics of the Spotted Stem Borer (*Chilo partellus*) in Contrasting Maize Farming Systems Under Variable Climatic Conditions in East Africa

**DOI:** 10.1155/sci5/7137270

**Published:** 2026-10-21

**Authors:** Chrysantus M. Tanga, Edward N. Karanja, Stephen Wainaina, Thomas Dubois, Nancy Mwende, Anthony Waititu, Samuel Mwalili, Noah Adamtey, Felix Matheri, Edwin Mwangi, Daisy Salifu

**Affiliations:** ^1^ International Centre of Insect Physiology and Ecology (icipe) Nairobi Kenya icipe.org; ^2^ Jomo Kenyatta University of Agriculture and Technology (JKUAT) Nairobi Kenya jkuat.ac.ke; ^3^ International Water Management Institute (IWMI) Accra Ghana

**Keywords:** Bayesian modeling, *Chilo partellus*, climatic variability, East Africa, integrated pest management, long-term farming systems, spotted maize stem borer, time-series analysis

## Abstract

The spotted stem borer (*Chilo partellus*) is one of the most destructive pests of maize in sub‐Saharan Africa, causing substantial yield losses and threatening food security among smallholder farming communities. This study investigated the long‐term relationships between climatic variability and *C. partellus* incidence across contrasting organic and conventional farming systems in two agroecological zones. Biweekly monitoring observations of *C. partellus* incidence were summarized quarterly for temporal analyses across four long‐term farming systems at Chuka and Thika: conventional high‐, organic high‐, conventional low‐, and organic low‐input. The resulting analytical series represented 32 and 28 quarterly positions at Chuka and Thika, respectively. Climatic variables (temperature, rainfall, and relative humidity) were obtained from on‐site weather stations. Spearman’s rank correlation, cross‐correlation, ARIMA/SARIMA time‐series models with exogenous climatic predictors (ARIMAX/SARIMAX), and Bayesian generalized linear mixed models were used to evaluate climatic associations and farming systems effects. Temperature emerged as the climatic variable most consistently associated with *C. partellus* incidence, showing significant negative relationships across all farming systems at Chuka and within the organic farming systems at Thika, whereas rainfall and relative humidity exhibited comparatively weak associations. Cross‐correlation analyses indicated predominantly contemporaneous and short lag associations (0–2 quarters) between climatic variables and pest incidence. Time‐series models were evaluated using descriptive measures of in‐sample agreement between observed and fitted values. Across farming systems, *R* ^2^ values ranged from 0.039 to 0.650, indicating substantial variation in the degree to which the fitted models represented the observed temporal patterns. These measures describe in‐sample model fit and should not be interpreted as evidence of out‐of‐sample predictive or forecasting performance. Bayesian analyses further indicated that *C. partellus* incidence was higher in the organic than in the conventional high‐input systems after accounting for climatic variability, whereas the 95% credible intervals for farming system effects in the low‐input systems overlapped zero. Overall, these findings suggest that climatic variability and farming system management are associated with long‐term variation in *C. partellus* incidence. The findings may inform the development of climate‐informed pest‐monitoring and integrated pest management strategies, while independently validated models would be required before application for pest forecasting under sustainable maize production in East Africa.

## 1. Introduction

Maize is one of the most important food and cash crops in Kenya and across sub‐Saharan Africa. However, its production is constrained by several insect pests, among which stem borers are particularly damaging [1]. The lepidopteran stem borers *Chilo partellus* (Swinhoe), an invasive species originating from Asia, and *Busseola fusca* (Fuller), a species native to Africa, are among the most economically important maize pests on the continent [2]. In eastern Africa, *C. partellus* is often the dominant species and is responsible for substantial yield losses. The larvae feed on maize leaves and stems, causing dead hearts, stem tunneling, and reduced grain yield. In Kenya, stem‐borer damage is estimated to cause annual maize yield losses of approximately 13.5%, valued at about US$80 million, of which *C. partellus* accounts for nearly US$23 million [2].

The occurrence and severity of stem‐borer infestations are influenced by several factors, including climatic conditions, farming practices, crop variety, and soil fertility [3–6]. Previous studies have shown that temperature, rainfall, and humidity affect the distribution, abundance, and seasonal dynamics of *C. partellus* [7, 8]. For example, research in Zimbabwe demonstrated that climatic conditions influence the distribution and incidence of *C. partellus* and *B. fusca*, with each species showing preferences for particular ecological zones [7]. Understanding how climatic factors influence pest populations is becoming increasingly important as climate variability continues to affect agricultural production systems. Improved understanding of climatic associations with pest dynamics may contribute to the development of pest‐monitoring and forecasting approaches and help inform the timing of pest management interventions [9]. Consequently, numerous studies have examined relationships between *C. partellus* populations and weather variables such as temperature, rainfall, and relative humidity [8, 10–14]. Although climatic factors have been associated with pest outbreaks, the strength and direction of these relationships vary among locations and production systems [15]. This variability highlights the importance of understanding location‐specific relationships between climatic conditions and pest dynamics when developing climate‐informed pest management and forecasting approaches.

Long‐term farming systems provide a unique opportunity to investigate how contrasting management practices modify pest responses to climatic variability. Organic and conventional systems differ in nutrient management, crop protection practices, and soil fertility management, which may directly or indirectly influence pest dynamics [16]. Understanding whether climatic effects differ between farming systems is important for developing resilient pest management strategies under climate change.

Time‐series approaches such as autoregressive integrated moving‐average (ARIMA) and seasonal autoregressive integrated moving‐average (SARIMA) models have been widely used to analyze and forecast biological and environmental processes. These models are particularly useful because they account for temporal dependence within data and can incorporate external predictors such as weather variables. Previous studies have successfully applied ARIMA‐based models to predict pest and disease dynamics, demonstrating their usefulness as early‐warning tools [15]. However, models intended for operational forecasting require appropriate independent or out‐of‐sample validation before their predictive performance can be established. Unlike previous studies that primarily examined climatic predictors of *C. partellus* outbreaks, the present study combines long‐term climatic records with contrasting organic and conventional farming systems and evaluates whether farming system effects remain after accounting for climatic, seasonal, and interannual variability using Bayesian mixed‐effects models in East Africa. Therefore, the present study examined the temporal relationships between *C. partellus* incidence and climatic variables across contrasting organic versus conventional farming systems at Chuka and Thika using the available long‐term monitoring observations. The study further developed and assessed ARIMA/SARIMA time‐series models with exogenous climatic predictors for describing temporal patterns in *C. partellus* incidence and evaluated farming system effects after accounting for climatic variability. Improved understanding of these relationships can support the development of climate‐informed pest‐monitoring and integrated pest management strategies and contribute to more sustainable maize production systems.

## 2. Materials and Methods

### 2.1. Study Site

The study was conducted at the Chuka and Thika sites in Kenya as part of a long‐term farming system comparative trial, which was established in 2007 (SysCom; https://www.system-comparison.fibl.org) [16]. The establishment date of the long‐term experiment should be distinguished from the temporal coverage of the *C. partellus* monitoring observations analyzed in the present study. The two sites are characterized by two cropping seasons, a long rain cropping season (March to July) and a short rain cropping season (October to December). Thika is situated in a semihumid ecological zone within Murang’a County with an annual precipitation of 900‐1100 mm and a mean annual temperature of 20°C. The Thika trial is located on Kenya Agricultural and Livestock Research Organization (KALRO) Horticultural Research Institute premises at Kandara [1°00.231′ S, 37°04.747′ E] and 1548 m above sea level). The Chuka trial is located 1500 m above sea level within subhumid agroecological zone in Tharaka Nithi County with an annual precipitation of 1500‐2400 mm and an annual mean temperature of 20°C. The Chuka trial is located on Kiereni primary school premises (0°20.864′ S, 37°38.792′ E).

### 2.2. Experimental Treatments and Management

The long‐term experiment consists of two farming systems (conventional and organic) at two input levels (high and low). Organic farming systems relied on organically approved nutrient inputs (compost, tithonia tea, mulch, and rock phosphate) together with biological and botanical pest management, whereas conventional systems relied primarily on synthetic mineral fertilizers and synthetic pesticides. The high‐input levels were designed to simulate farming practices used by growers targeting distant high‐value markets, both locally and globally, with the support of irrigation. The low‐input levels, on the other hand, were designed to reflect the typical use of pesticides and fertilizers by smallholder farmers, whose produce is primarily consumed at home or sold in local markets.

The experimental design included four replications of randomized complete blocks with four farming systems (conventional high, conventional low, organic high, and organic low). Experimental plots measured 8 × 8 m with a net plot of 6 × 6 m for data collection separated by a 2‐m buffer zone of barren ground. Over the course of the experiment, maize and baby corn crops were planted consecutively during long rain seasons, ensuring continuous data collection for the whole season. During the short rain seasons, cabbages, French beans, and potatoes were planted in high‐input systems and kales, spinach, common beans, and potatoes in the low‐input systems within a 3‐year crop rotation.

### 2.3. Pest Incidence Scouting and Weather Data

Biweekly data collection on pest incidence commenced when the crops were at three‐leaf stage and ended at crop maturity. Forty plants were randomly sampled from each plot upon which *C. partellus* incidence was recorded. Biweekly pest‐incidence assessments were conducted within the replicated experimental plots using the same sampling protocol throughout the available monitoring periods to ensure consistency of the field observations. Thus, the original field data represented repeated assessments within the replicate plots of each farming system. For the subsequent temporal analyses, these repeated monitoring observations were summarized by a farming system and calendar quarter to generate quarterly *C. partellus* incidence observations. Accordingly, plot‐level observations constituted the underlying field measurements, whereas farming‐system‐by‐quarter observations constituted the analytical observations used for the time‐series analyses. Daily average temperatures, relative humidity, and total rainfall data were recorded from automatic weather stations installed at experimental sites. For the temporal analyses, the weather observations were summarized to correspond with the quarterly temporal scale used for *C. partellus* incidence.

For descriptive interpretation of temporal pest pressure, a 20% incidence reference level was included in the incidence plots. A 20% plant infestation level has been used as an action threshold for lepidopteran pest management in maize, although recommended thresholds vary with pest species, crop developmental stage, damage metric, and production context [17]. Studies on cereal stem borers in Kenyan maize have similarly demonstrated strong relationships between stem‐borer density and crop yield loss [18]. Accordingly, the 20% line used in the present study serves only as a comparative incidence reference and should not be interpreted as a formally established economic injury or action threshold specifically for *C. partellus*.

### 2.4. Data Analysis

#### 2.4.1. Exploratory Analysis

For the temporal analyses, the repeated field‐monitoring observations were summarized at the farming system and quarterly level. The resulting analytical datasets comprised 124 and 112 observations, representing 32 and 28 quarterly positions in Chuka and Thika, respectively. Meteorological variables (temperature, relative humidity, and rainfall) and *C. partellus* incidence data from the four farming systems at Chuka and Thika were explored graphically using the *ggplot2* package in R [19]. Associations between climatic variables and *C*. *partellus* incidences were initially examined using Spearman’s rank correlation coefficients.

To investigate delayed relationships between weather conditions and *C. partellus* incidence, cross‐correlation analysis was performed using the ccf (*x*, *y*) function in R [20], with *C. partellus* incidence supplied as the first argument (*x*) and each weather variable supplied as the second argument (*y*), i.e., ccf (stem‐borer incidence, weather variable). With this argument order, the coefficient at lag *k* represents the correlation between stem‐borer incidence at time *t + k* and the weather variable at time *t*. Therefore, a positive lag indicates that the weather observation preceded the associated stem‐borer incidence observation, whereas a negative lag indicates that the stem‐borer incidence observation preceded the corresponding weather observation. A lag of zero represents a contemporaneous association within the same quarter. Positive and negative cross‐correlation coefficients indicate the direction of the association, whereas the lag indicates its temporal ordering. These relationships were interpreted as temporal associations and not as evidence of causation.

#### 2.4.2. Time‐Series Modeling

Time‐series models were used to examine temporal patterns in *C*. *partellus* incidence and to evaluate the contribution of climatic variables to changes in infestation over time. ARIMA models were fitted as they can account for temporal dependence in observations and are widely used for analyzing ecological and agricultural time‐series data [21].

Model fitting was performed using the *forecast* package in R [22]. The *auto.arima*() function was used to identify candidate ARIMA error structures, with the model selection based on the corrected Akaike information criterion (AICc). AICc was used because of the relatively small number of observations available for each farming system time series [23]. The ARIMA structure is denoted as ARIMA (*p, d, q*) (*P*, *D*, *Q*)_*s*_, where *p*, *d*, and *q* represent the nonseasonal autoregressive order, degree of differencing, and moving‐average order, respectively; *P*, *D*, and *Q* represent the corresponding seasonal orders; and *s* denotes the seasonal period, which was four for the quarterly series.

The orders of nonseasonal and differencing were determined using the default procedures implemented in *auto.arima*() function. The nonseasonal differencing order *d* was selected using the Kwiatkowski–Phillips–Schmidt–Shin (KPSS) unit‐root testing procedure, whereas the seasonal differencing order (*D*) was selected using the default seasonal strength procedure based on seasonal‐trend decomposition using LOESS (STL). The remaining autoregressive and moving‐average orders were evaluated by the model selection procedure, with candidate models compared using AICc [22].

For the time‐series analyses, the biweekly pest‐incidence observations were aggregated into calendar quarters. Observations were grouped as Quarter 1 (January–March), Quarter 2 (April–June), Quarter 3 (July–September), and Quarter 4 (October–December). Quarterly pest incidence was calculated as the mean of the available biweekly incidence observations within each 3‐month period for each farming system. This aggregation provided a common quarterly temporal scale for examining the seasonal variation in pest incidence and its associations with meteorological conditions.

Missing pest‐incidence values accounted for less than 5% of the biweekly observations. Observations with missing incidence values were excluded, and quarterly mean incidence was calculated using the available biweekly observations within each quarter. No data imputation was performed. Given the small proportion of missing observations, quarterly estimates were calculated from the available observations; the potential influence of missing observations should nevertheless be considered when interpreting the temporal analyses.

Prior to time‐series modeling, pest‐incidence proportions were logit‐transformed. Because the logit transformation is undefined for proportions equal to 0 or 1, values of 0 and 1 were replaced with 0.001 and 0.999, respectively. The transformed values were used for model fitting, and predictions were converted back to the original proportion scale using the inverse‐logit transformation. The transformed quarterly *C. partellus* incidence observations were analyzed separately for each farming system at Chuka and Thika using the available quarterly monitoring series described above. Both nonseasonal ARIMA models and seasonal ARIMA (SARIMA) models were evaluated. Seasonal models were fitted using a quarterly frequency (*s* = 4) to account for recurring seasonal patterns in *C. partellus* incidence within the analyzed quarterly series.

To assess associations between climatic variables and *C. partellus* incidence, average temperature, relative humidity, and rainfall were incorporated as exogenous variables. To minimize multicollinearity among temperature variables, average temperature was retained as the exogenous temperature variable in the final models, while minimum and maximum temperature were excluded because of their close correlation with average temperature. Two sets of models were fitted for each farming system. First, ARIMA or SARIMA models were fitted without climatic regressors to describe temporal and seasonal dependence in *C. partellus* incidence. Second, ARIMAX or SARIMAX models were fitted with climatic variables as external regressors. The ARIMA error structure was selected separately for each model; therefore, the selected orders differed between models fitted with and without climatic regressors. Candidate models were compared using AICc, and the model with the lowest AICc was selected for each farming system. The selected model structures, climatic variable coefficients, and model‐fit statistics are reported in Tables 1, 2, 3, and 4.

**TABLE 1:** Best‐fitting ARIMA error structures and model‐fit statistics for ARIMAX and SARIMAX models of quarterly *C. partellus* incidence under four farming systems at Chuka, with average temperature, relative humidity, and rainfall included as exogenous predictors.

Farming system	Model class	ARIMA error structure	AICc	RMSE	*Q* ^∗^ (*p* value)
Conventional high	ARIMAX	ARIMA (0, 1, 0)	123.29	1.491	7.487 (0.278)
Organic high	SARIMAX	ARIMA (0, 1, 1) (0, 0, 1)_4_	120.08	1.145	3.701 (0.448)
Conventional low	SARIMAX	ARIMA (0, 1, 1) (1, 0, 0)_4_	142.94	1.830	6.382 (0.172)
Organic low	SARIMAX	ARIMA (0, 1, 1) (0, 0, 1)_4_	136.61	1.555	5.384 (0.250)

*Note:* ARIMAX = autoregressive integrated moving‐average model with exogenous predictors; SARIMAX = seasonal autoregressive integrated moving‐average model with exogenous predictors; AICc = Akaike information criterion corrected for small sample size; and *Q* ^∗^ = Ljung–Box statistic used to test for residual autocorrelation, with the corresponding *p* value reported in parentheses. A Ljung–Box *p* value > 0.05 indicates no statistically significant residual autocorrelation. Subscript 4 denotes quarterly seasonality.

Abbreviation: RMSE = root‐mean‐square error.

**TABLE 2:** Coefficient estimates for the ARIMAX and SARIMAX models incorporating average temperature, relative humidity, and rainfall as exogenous predictors of quarterly *C*. *partellus* incidence at Chuka.

Model term	Estimate	Std. error	*z* value	Pr (> |*z*|)
Conventional high: ARIMAX, ARIMA (0, 1, 0)				
Average temperature (°C)	−0.028	0.074	−0.372	0.7097
Relative humidity (%)	0.028	0.019	1.48	0.1395
Rainfall (mm)	0.009	0.019	0.46	0.6447
Organic high: SARIMAX, ARIMA (0, 1, 1) (0, 0, 1)_4_				
MA (1)	−0.700	0.103	−6.68	< 0.0001
SMA (1)	0.939	0.829	4.37	0.2575
Average temperature (°C)	−0.118	0.063	−1.03	0.0592
Relative humidity (%)	0.043	0.013	1.12	0.0008
Rainfall (mm)	0.021	0.024	−1.16	0.3786
Conventional low: SARIMAX, ARIMA (0, 1, 1) (1, 0, 0)_4_				
MA (1)	−0.656	0.179	−3.671	0.0002
SAR (1)	0.371	0.191	1.946	0.0517
Average temperature (°C)	−0.800	0.093	−0.854	0.3930
Relative humidity (%)	0.001	0.019	0.053	0.9579
Rainfall (mm)	0.018	0.040	0.460	0.6457
Organic low: SARIMAX, ARIMA (0, 1, 1) (0, 0, 1)_4_				
MA (1)	−0.371	0.249	−1.492	0.1356
SMA (1)	0.839	0.710	1.180	0.2379
Average temperature (°C)	−0.084	0.091	−0.919	0.3579
Relative humidity (%)	0.007	0.017	0.391	0.6956
Rainfall (mm)	0.028	0.039	0.720	0.4717

*Note:* MA (1) = first‐order nonseasonal moving‐average term; SMA (1) = first‐order seasonal moving‐average term. Subscript 4 in the SARIMAX specification denotes quarterly seasonality. Estimate = estimated model coefficient; std. error = standard error of the coefficient.

**TABLE 3:** Best‐fitting ARIMA error structures and model‐fit statistics of quarterly *C*. *partellus* incidence under four farming systems at Thika, with average temperature, relative humidity, and rainfall included as exogenous predictors.

Farming system	Model class	ARIMA error structure	AICc	RMSE	*Q* ^∗^ (*p* value)
Conventional high	ARIMAX	ARIMA (0, 0, 0)	124.41	1.875	4.411 (0.620)
Organic high	SARIMAX	ARIMA (3, 1, 0) (1, 0, 0)_4_	122.22	1.364	1.318 (0.723)
Conventional low	SARIMAX	ARIMA (1, 1, 0) (1, 0, 0)_4_	121.86	1.630	0.757 (0.944)
Organic low	ARIMAX	ARIMA (0, 1, 1)	118.26	1.655	4.165 (0.526)

*Note:* ARIMAX = autoregressive integrated moving‐average model with exogenous predictors; SARIMAX = seasonal autoregressive integrated moving‐average model with exogenous predictors; AICc = Akaike information criterion corrected for small sample size; and *Q* ^∗^ = Ljung–Box statistic used to test for residual autocorrelation, with the corresponding *p* value reported in parentheses. A Ljung–Box *p* value > 0.05 indicates no statistically significant residual autocorrelation. Subscript 4 denotes quarterly seasonality.

Abbreviation: RMSE = root‐mean‐square error.

**TABLE 4:** Coefficient estimates for the ARIMAX and SARIMAX models incorporating temperature, relative humidity and rainfall as exogenous predictors of *C*. *partellus* incidence at Thika.

Model term	Estimate	Std. error	*z* value	Pr (> |*z*|)
Conventional high: ARIMAX, ARIMA (0, 0, 0)				
Average temperature (°C)	−0.262	0.074	−3.548	0.0004
Relative humidity (%)	0.017	0.025	0.687	0.4922
Rainfall (mm)	0.011	0.032	0.348	0.72768
Organic high: SARIMAX, ARIMA (3, 1, 0) (1, 0, 0)_4_				
AR (1)	−0.282	0.161	−1.757	0.0789
AR (2)	−0.484	0.156	−3.100	0.0019
AR (3)	−0.735	0.206	−3.568	0.0004
SAR (1)	−0.654	0.171	−3.819	0.0001
Average temperature (°C)	−0.520	0.140	−3.706	0.0002
Relative humidity (%)	0.014	0.025	0.588	0.5563
Rainfall (mm)	0.018	0.023	0.790	0.4298
Conventional low: SARIMAX, ARIMA (1, 1, 0) (1, 0, 0)_4_				
AR (1)	−0.689	0.155	−4.436	< 0.0001
SAR (1)	−0.521	0.192	−2.711	0.0067
Average temperature (°C)	−0.191	0.116	−1.644	0.1002
Relative humidity (%)	0.033	0.022	1.469	0.1418
Rainfall (mm)	−0.023	0.024	−0.928	0.3533
Organic low: ARIMAX, ARIMA (0, 1, 1)				
MA (1)	−0.663	0.138	−4.814	< 0.0001
Average temperature (°C)	−0.067	0.152	−0.453	0.6503
Relative humidity (%)	0.018	0.025	0.701	0.4836
Rainfall (mm)	−0.029	0.027	−1.086	0.2777

*Note:* AR (1), AR (2), and AR (3) denote the first‐, second‐, and third‐order nonseasonal autoregressive terms, respectively; SAR (1) = the first‐order seasonal autoregressive term, MA (1) = first‐order nonseasonal moving‐average term. Subscript 4 in the SARIMAX specification denotes quarterly seasonality. Estimate = estimated model coefficient; std. error = standard error of the coefficient.

#### 2.4.3. Model Diagnostics

After identifying the optimal model, diagnostic checks on residuals were conducted to ensure the adequacy of the model fit. These included, autocorrelation function (ACF) and partial autocorrelation function (PACF) plots of residuals to assess for any remaining autocorrelation, histogram of residuals to verify the assumption of normality, and Ljung–Box test to test for randomness of residuals at different lags. Models with nonsignificant Ljung–Box tests were considered to show no evidence of remaining residual autocorrelation. These diagnostics were used to assess in‐sample model adequacy and not out‐of‐sample predictive performance.

#### 2.4.4. Bayesian Analysis of Farming System Effects

To determine differences in *C. partellus* incidence between organic and conventional farming systems while accounting for climatic and temporal variation, Bayesian generalized linear mixed models were fitted using the *brms* package in R. The response variable was the number of stems infested by *C*. *partellus* relative to the total number of stems assessed per plot, modeled using a binomial distribution with a logit link function.

Because the high‐ and low‐input systems differed in management intensity, irrigation regime, and crop rotations, comparisons were restricted to farming systems within the same input level. Consequently, separate models were fitted for organic high versus conventional high and organic low versus conventional low systems at each study site. Fixed effects included farming system (organic versus conventional), climatic variables (temperature, relative humidity, and rainfall), and quarter to account for seasonal variation. Year was included as a random intercept to account for interannual variation. Plot identity was not included as an additional random effect; consequently, repeated observations within individual experimental plots were not explicitly represented through a plot‐level random effect. Farming system effects from these models were therefore interpreted with this model structure in consideration. Alternative models containing current and lagged climatic variables were evaluated, and model performance was compared using leave‐one‐out (LOO) cross‐validation. The model with the lowest leave‐one‐out information criterion (LOOIC) was selected for inference. Posterior summaries are reported as posterior means and 95% credible intervals (CrIs). Effects were considered supported when the 95% CrI did not overlap zero. Posterior expected infestation probabilities for each farming system were obtained to facilitate the biological interpretation of treatment effects.

The dataset was examined for completeness prior to analysis. A very small number of observations contained missing values and were excluded from the analyses. No data imputation was performed.

Agreement between the observed and ARIMA‐fitted values was assessed using the coefficient of determination (R^2^) and root‐mean‐square error (RMSE). *R* ^2^ was calculated from a simple linear regression of the back‐transformed fitted values on the observed incidence values, both expressed on the original percentage scale. RMSE was obtained from the fitted ARIMAX/SARIMAX models and is reported on the logit‐transformed scale.

Because R^2^ is not a standard goodness‐of‐fit measure for differenced ARIMA models, it was used only as a descriptive measure of in‐sample agreement and not for model selection. Model selection was based on AICc, while model adequacy was assessed using residual diagnostics and the Ljung–Box test. All analyses were implemented using R, Version 4.3.1 [24].

## 3. Results

### 3.1. Temporal Trends in *C. partellus* Incidence and Climatic Variables

Temporal patterns of *C. partellus* incidence were broadly similar across the four farming systems (conventional high, organic high, conventional low, and organic low) at both Chuka and Thika (Figures 1–3). Although the timing and magnitude of infestations varied slightly among farming systems and between the two agroecological zones, the overall temporal pattern was consistent. At both sites, *C. partellus* incidence was highest during the establishment phase of the long‐term experiment and declined markedly thereafter. In Chuka, the first major infestation peak occurred during the third and fourth quarters of the first year (Figure 1A), whereas in Thika, the highest infestation was recorded during the first quarter of the second year (Figure 1B). Following these initial outbreaks, *C. partellus* incidence declined and remained relatively low across all farming systems for most of the study period.

**FIGURE 1:**
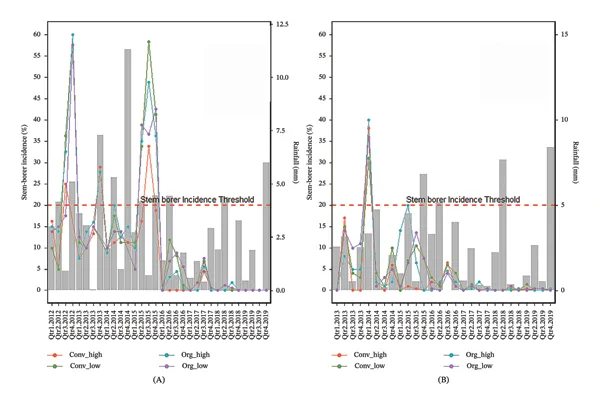
Temporal relationship between rainfall and stem‐borer (*C. partellus*) incidence in long‐term farming systems at Chuka (A) and Thika (B). Continuous lines represent the quarterly *C. partellus* incidence across four farming systems; gray bars indicate quarterly rainfall amounts. The horizontal dashed red line indicates the 20% incidence reference level used to facilitate the visual comparison of the temporal variation in *C. partellus* incidence; it does not represent a formally established economic injury or action threshold for *C. partellus*.

**FIGURE 2:**
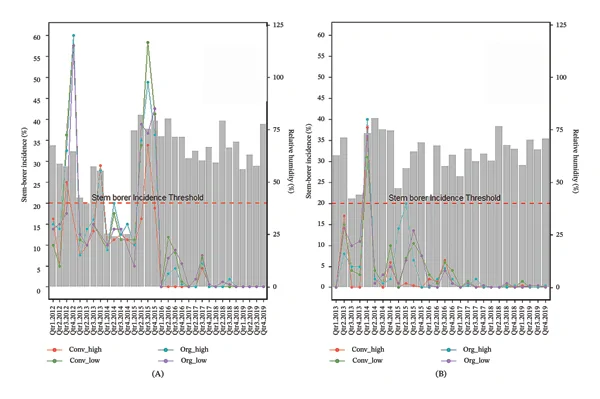
Temporal relationship between relative humidity and stem‐borer (*C. partellus*) incidence in long‐term farming systems at Chuka (A) and Thika (B). Continuous lines represent the quarterly *C. partellus* incidence across four farming systems; gray bars indicate quarterly relative humidity levels. The horizontal dashed red line indicates the 20% incidence reference level used to facilitate the visual comparison of the temporal variation in *C. partellus* incidence; it does not represent a formally established economic injury or action threshold for *C. partellus*.

**FIGURE 3:**
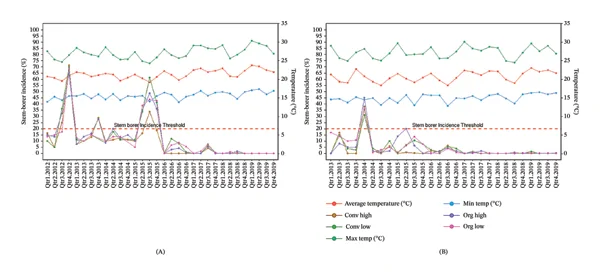
Temporal relationship between temperature and stem‐borer (*C. partellus*) incidence in long‐term farming systems at Chuka (A) and Thika (B). Continuous lines represent the quarterly *C. partellus* incidence across four farming systems. The horizontal dashed red line indicates the 20% incidence reference level used to facilitate the visual comparison of the temporal variation in *C. partellus* incidence; it does not represent a formally established economic injury or action threshold for *C. partellus*.

Rainfall exhibited considerable seasonal and interannual variations at both sites throughout the study (Figure 1). The highest *C. partellus* infestations generally occurred during the early years of the experiment when rainfall variability was greatest. However, in subsequent years, *C. partellus* incidence remained consistently low despite continued fluctuations in rainfall. Similarly, relative humidity showed a clear seasonal variation at both locations (Figure 2). In Chuka, the elevated *C. partellus* incidence during the first 2 years of the study coincided with periods of fluctuating humidity (Figure 2A). Similarly, in Thika, the early infestation peak occurred during periods of relatively high humidity (Figure 2B). Thereafter, humidity continued to fluctuate seasonally while *C. partellus* incidence remained low across farming systems.

Temperature also exhibited clear seasonal variation throughout the study period (Figure 3). At both Chuka and Thika, the highest *C. partellus* incidence occurred during the early years of the experiment when greater fluctuations in minimum, average, and maximum temperatures were observed. Despite continued seasonal variation in temperature during later years, *C. partellus* incidence remained below the 20% reference level during most subsequent observations across all farming systems.

The temporal patterns indicate that the largest *C. partellus* outbreaks occurred during the early part of the analyzed time series, after which incidence generally remained low across farming systems at both agroecological zones.

### 3.2. Relationship Between *C. partellus* Incidence and Climatic Variables Across Farming Systems

Spearman correlation analysis suggested that temperature was the climatic variable most consistently associated with *C. partellus* incidence across farming systems at both Chuka and Thika (Table 5). In general, *C. partellus* incidence tended to decrease with increasing temperature, although the strength of these relationships varied among farming systems and between the two agroecological zones.

**TABLE 5:** Spearman correlation coefficients between quarterly average climatic variables and *C. partellus* incidence across different farming systems at Chuka and Thika.

Site	Incidence in farming system	Average relative humidity (%)	Minimum temperature (°C)	Average temperature (°C)	Maximum temperature (°C)	Rainfall (mm)
Thika	Conventional high	0.0830	−0.2188	−0.3486	−0.1444	0.2537
	*p* value	0.6747	0.2633	0.0691	0.4636	0.1928
	Organic high	−0.1138	−0.4830	−0.4366	−0.2767	0.0075
	*p* value	0.5644	**0.0092**	**0.0202**	0.1540	0.9698
	Conventional low	0.0872	−0.1868	−0.3397	−0.1918	0.2055
	*p* value	0.659	0.3412	0.0770	0.3281	0.2942
	Organic low	0.0008	−0.4524	−0.4098	−0.1999	0.0268
	*p* value	0.9966	**0.0157**	**0.0303**	0.3077	0.8922

Chuka	Conventional high	−0.0790	−0.4127	−0.4529	−0.4096	0.3302
	*p* value	0.6675	**0.0189**	**0.0092**	**0.0199**	0.0649
	Organic high	0.0651	−0.4121	−0.4937	−0.4432	0.3004
	*p* value	0.7232	**0.01191**	**0.0041**	**0.0111**	0.0948
	Conventional low	0.0653	−0.6062	−0.6786	−0.6407	0.2292
	*p* value	0.7318	**0.0004**	**< 0.0001**	**0.0001**	0.2232
	Organic low	0.0857	−0.6809	−0.7839	−0.7281	0.1714
	*p* value	0.6524	**< 0.0001**	**< 0.0001**	**< 0.0001**	0.3650

*Note:* Significant correlations (*p* < 0.05) are shown in bold.

At Chuka, minimum, average and maximum temperatures were negatively correlated with *C. partellus* incidence across all farming systems. The strongest relationships were observed in the low‐input systems, particularly the organic low‐input system, where correlation coefficients ranged from −0.68 to −0.78 (*p* < 0.001). Similarly, significant negative correlations were recorded in the conventional low‐input system (*r* = −0.61 to −0.68, *p* < 0.001). Moderate but significant negative correlations were also observed in the high‐input systems (*r* = −0.41 to −0.49, *p* < 0.05). Similarly, at Thika, negative correlations between temperature and *C. partellus* incidence were also observed, although they were generally weaker and less consistent than those recorded at Chuka. Significant associations were primarily detected in the organic systems, where minimum and average temperatures were negatively correlated with infestation in both organic high‐input (*r* = −0.48 and −0.44, respectively; *p* < 0.05) and organic low‐input systems (*r* = −0.45 and −0.41, respectively; *p* < 0.05). Relative humidity showed weak and nonsignificant correlations with *C. partellus* incidence across all farming systems at both sites. Similarly, rainfall was not significantly correlated with *C. partellus* incidence, although positive correlations were observed in several farming systems. Overall, these results suggest that temperature is more associated with variation in *C. partellus* incidence than rainfall or relative humidity during the study period.

### 3.3. Cross‐Correlation Analysis

Cross‐correlation analysis revealed site‐specific and farming system–specific associations between *C. partellus* incidence and meteorological variables (Figure 4). At Chuka, *C. partellus i*ncidence was most consistently associated with temperature variables. Under both conventional high‐input (Figure 4A) and organic high‐input systems (Figure 4B), positive correlations were observed between *C. partellus* incidence and minimum and average temperatures at contemporaneous and short positive‐lag associations ranging from 0 to +2 quarters. Given the order of the variables in the ccf() analysis, positive lags indicate that temperature observations preceded the associated stem‐borer incidence observations by up to two‐quarters. Lag 0 represents a contemporaneous association. Associations between *C. partellus* incidence and rainfall or relative humidity at Chuka were generally weaker and less consistent across lags. In the low‐input systems (Figure 4C,D), cross‐correlations were generally weaker than those observed in the high‐input systems, although positive correlations between average temperature and *C. partellus* incidence were evident at lag 0 and +1 quarter in the conventional low‐input system.

**FIGURE 4:**
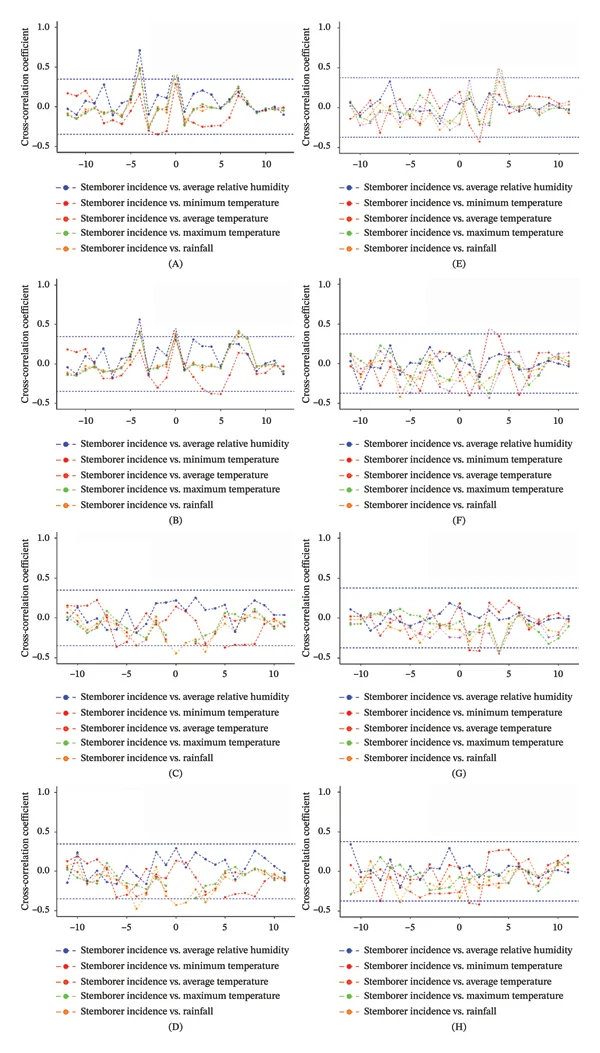
Cross‐correlation functions (CCFs) between climatic variables and stem‐borer (*C. partellus*) incidence at Chuka (left column) and Thika (right column). Panels (A) and (E) represent the conventional high‐input system, (B) and (F) the organic high‐input system, (C) and (G) the conventional low‐input system, and (D) and (H) the organic low‐input system. Blue dashed horizontal lines indicate the approximate 95% confidence limits. Cross‐correlation coefficients extending beyond these limits are considered statistically significant.

At Thika, the cross‐correlation patterns differed from those observed at Chuka. In the conventional high‐input system (Figure 4E), *C. partellus* incidence was negatively correlated with rainfall and relative humidity at several positive lags, while minimum temperature showed positive correlations at short lags. In contrast, the organic high‐input and low‐input systems (Figure 4F–H) exhibited relatively weak associations between *C. partellus* incidence and the climatic variables across most lag periods.

### 3.4. Autocorrelation and Partial Autocorrelation Analyses

ACF and PACF analyses indicated generally weak temporal dependence in *C. partellus* incidence across farming systems at both Chuka and Thika (Figures 5 and 6). In most cases, autocorrelation coefficients declined rapidly with increasing lag, and the majority of ACF and PACF values remained within the 95% confidence limits, indicating limited persistence in *C. partellus* incidence over time. At Chuka (Figure 5), the conventional high‐input and organic high‐input systems exhibited small positive autocorrelations at short lags, particularly around lag 1. Similar but weaker patterns were observed in the conventional low‐input system. The organic low‐input system showed little evidence of temporal dependence, with most coefficients remaining close to zero across all lags. Similarly, the PACF plots displayed only a few isolated significant spikes, suggesting a limited autoregressive structure. Comparable patterns were observed at Thika (Figure 6). Small positive autocorrelations were evident at short lags in the conventional high‐ and conventional low‐input systems, whereas both organic systems exhibited weak autocorrelation throughout the study period. The corresponding PACF plots likewise showed only a small number of significant spikes and no persistent autoregressive structure.

**FIGURE 5:**
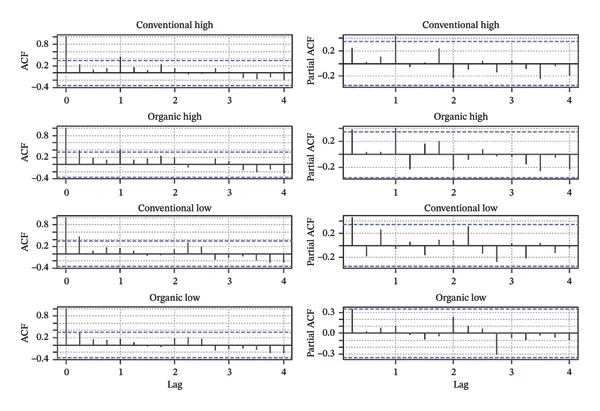
Autocorrelation function (ACF) and partial autocorrelation function (PACF) plots of quarterly stem‐borer (*C. partellus*) incidence in the conventional high‐input, organic high‐input, conventional low‐input, and organic low‐input farming systems at Chuka. Blue dashed lines indicate the approximate 95% confidence limits. Bars extending beyond the confidence limits indicate statistically significant temporal autocorrelation.

**FIGURE 6:**
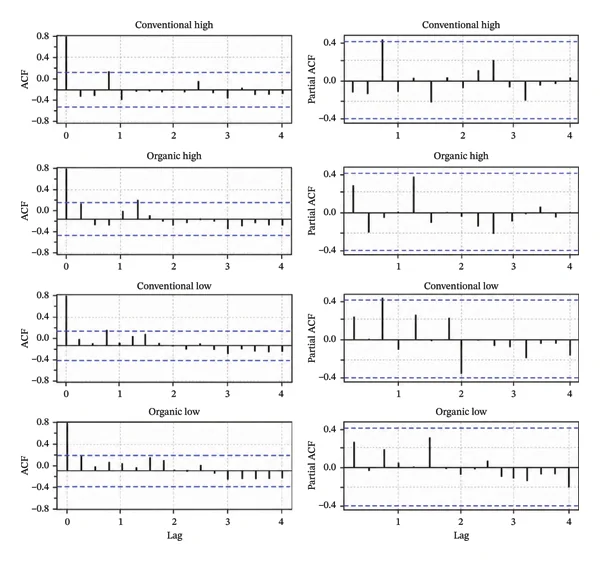
Autocorrelation function (ACF) and partial autocorrelation function (PACF) plots of quarterly stem borer (*C. partellus*) in the conventional high‐input, organic high‐input, conventional low‐input, and organic low‐input farming systems at Thika. Blue dashed lines indicate the approximate 95% confidence limits. Bars extending beyond the confidence limits indicate statistically significant temporal autocorrelation.

### 3.5. ARIMA Modeling of *C. partellus* Incidence at Chuka and Thika

The ARIMAX and SARIMAX models identified differences in the temporal patterns of *C. partellus* incidence across farming systems and study sites (Tables 1, 2, 3, and 4). At Chuka, seasonal models provided the best fit for three of the four farming systems, namely, the organic high‐input, conventional low‐input, and organic low‐input systems (Table 1). The conventional high‐input system was best described by a nonseasonal ARIMAX model. At Thika, seasonal models provided the best fit for the organic high‐input and conventional low‐input systems, whereas nonseasonal models were selected for the conventional high‐input and organic low‐input systems (Table 3). The conventional high‐input system at Thika was described by an ARIMAX model with an ARIMA (0, 0, 0) error structure, indicating that the climatic predictors were included without additional autoregressive, differencing or moving‐average terms. Among the climatic variables, relative humidity was positively associated with *C. partellus* incidence in the organic high‐input system at Chuka (*p* = 0.0008). Average temperature, relative humidity, and rainfall were not significantly associated with incidence in the other farming systems at Chuka (Table 2). At Thika, average temperature was negatively associated with *C. partellus* incidence in the conventional high‐input (*p* = 0.0004) and organic high‐input (*p* = 0.0002) systems (Table 4). No significant climatic effects were detected in the conventional low‐input or organic low‐input systems at Thika. Rainfall was not significantly associated with *C. partellus* incidence in any farming system at either site.

The autoregressive and seasonal parameters included in several of the selected models were significant, indicating that previous observations and seasonal patterns contributed to explaining variation in *C. partellus* incidence. However, the strength and direction of climatic effects differed among farming systems and agroecological zones, suggesting that local environmental conditions and management practices may modify the response of *C. partellus* populations to temperature.

### 3.6. Model Diagnostics and Goodness of Fit

The adequacy of the fitted ARIMA models was assessed using residual diagnostics based on the Ljung–Box test (Tables 1 and 3). The Ljung–Box tests were nonsignificant for all fitted models (*p* > 0.05), indicating no evidence of remaining residual autocorrelation. Agreement between the observed and fitted values varied among farming systems and sites (Table 6). At Chuka, the organic high‐input system showed the highest in‐sample agreement (*R* ^2^ = 0.650), followed by the organic low‐input (*R* ^2^ = 0.522), conventional high‐input (*R* ^2^ = 0.410), and conventional low‐input (*R* ^2^ = 0.250) systems. At Thika, in‐sample agreement was highest for the organic high‐input system (*R* ^2^ = 0.494), followed by the conventional low‐input (*R* ^2^ = 0.357), organic low‐input (*R* ^2^ = 0.272), and conventional high‐input (*R* ^2^ = 0.039) systems. Overall, the models represented the observed temporal patterns to varying degrees across farming systems, and these in‐sample measures should not be interpreted as evidence of out‐of‐sample predictive or forecasting performance.

**TABLE 6:** Descriptive measures of in‐sample agreement between observed and fitted *C. partellus* incidence for the ARIMAX and SARIMAX models fitted to *C. partellus* incidence under different farming systems at Chuka and Thika.

Farming system	Model class	ARIMA error structure	*R* ^2^	RMSE
*Chuka*				
Conventional high	ARIMAX	ARIMA (0, 1, 0)	0.410	1.491
Organic high	SARIMAX	ARIMA (0, 1, 1) (0, 0, 1)_4_	0.650	1.145
Conventional Low	SARIMAX	ARIMA (0, 1, 1) (1, 0, 0)_4_	0.250	1.830
Organic low	SARIMAX	ARIMA (0, 1, 1) (0, 0, 1)_4_	0.522	1.555

*Thika*				
Conventional high	ARIMAX	ARIMA (0, 0, 0)	0.039	1.875
Organic high	SARIMAX	ARIMA (3, 1, 0) (1, 0, 0)_4_	0.494	1.364
Conventional low	SARIMAX	ARIMA (1, 1, 0) (1, 0, 0)_4_	0.357	1.630
Organic low	ARIMAX	ARIMA (0, 1, 1)	0.272	1.655

*Note:* *R* ^2^ was calculated from a linear regression of back‐transformed fitted incidence values on observed incidence values, both on the original percentage scale. Root‐mean‐square error (RMSE) was obtained directly from the fitted ARIMAX/SARIMAX models and is reported on the logit scale. *R* ^2^ and RMSE are on different scales and should therefore not be directly compared numerically. ARIMAX = autoregressive integrated moving‐average model with exogenous predictors; SARIMAX = seasonal autoregressive integrated moving‐average model with exogenous predictors. Subscript 4 denotes quarterly seasonality.

### 3.7. Observed and Fitted *C*. *partellus* Incidence

Visual comparison of observed and fitted values showed varying levels of in‐sample agreement across farming systems at both Chuka and Thika (Figures 7 and 8). The fitted ARIMA models represented some of the broad temporal variation in *C. partellus* incidence, including the general decline following the initial outbreak years. Agreement was generally better during periods of relatively stable pest incidence but weaker for some series. In particular, the models underestimated the magnitude of several outbreak peaks during the early years of the experiment, suggesting that abrupt short‐term fluctuations were less well represented than broader long‐term population trends.

**FIGURE 7:**
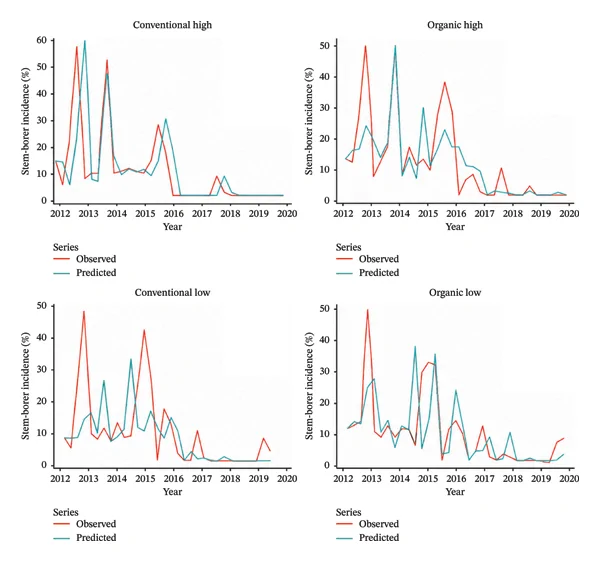
Observed and ARIMA‐fitted quarterly stem‐borer (*C. partellus*) incidence in the four farming systems at Chuka. The red line represents the observed incidence, while the blue line represents the fitted values from the selected ARIMAX/SARIMAX models.

**FIGURE 8:**
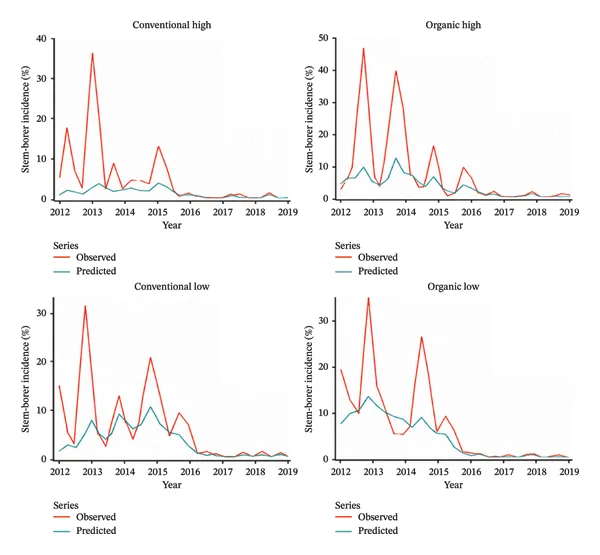
Observed and ARIMA‐fitted quarterly stem‐borer (*C. partellus*) incidence in the four farming systems at Thika. The red line represents the observed incidence, while the blue line represents the fitted values from the selected ARIMAX/SARIMAX models.

### 3.8. Effects of Farming System and Climatic Variables on *C*. *partellus* Incidence

Leave‐one‐out cross‐validation identified different best‐supported Bayesian generalized linear mixed models for the two agroecological zones. At Chuka, models incorporating current climatic variables provided the best fit, whereas models including lagged climatic variables received greater support for Thika. These models were subsequently used to estimate farming system effects while accounting for climatic covariates, seasonal effects, and interannual variation.

Within the high‐input systems, posterior estimates consistently indicated higher *C. partellus* incidence in the organic than in the conventional farming system at both Chuka (*β* = 0.34; 95% CrI: 0.15–0.52) and Thika (*β* = 1.02; 95% CrI: 0.72–1.33), as the corresponding CrIs excluded zero (Table 7). In contrast, no evidence of farming system differences was detected within the low‐input systems at either site because the 95% CrIs overlapped zero.

**TABLE 7:** Posterior estimates of farming system effects on *C*. *partellus* incidence from Bayesian generalized linear mixed models at Chuka and Thika.

Site	Input level	Estimate (*β*)	95% credible interval	Interpretation
Chuka	High	0.34	0.15, 0.52	Organic > conventional
Chuka	Low	−0.06	−0.25, 0.12	No difference
Thika	High	1.02	0.72, 1.33	Organic > conventional
Thika	Low	0.05	−0.02, 0.29	No difference

These findings indicate that differences between organic and conventional management were evident only under high‐input conditions after accounting for climatic variation, whereas comparable management effects were not observed under low‐input conditions. Complete posterior estimates for all model parameters are presented in Supporting Tables S1 and S2.

## 4. Discussion

This study investigated the influence of climatic variability on the long‐term dynamics of *C. partellus* incidence within contrasting organic and conventional maize farming systems at two agroecological zones in Kenya. Using the available long‐term pest‐incidence and climatic monitoring observations, together with time‐series and Bayesian modeling approaches, the analysis identified associations between temperature and *C. partellus* incidence across several farming systems. However, the strength and direction of these relationships differed between sites and farming systems. Furthermore, farming system differences persisted under high‐input management after accounting for the climatic variation, highlighting that both climatic conditions and management practices contributed to long‐term *C. partellus* incidence. These findings provide new insights into the interactions between climate and farming systems and have important implications for climate‐informed pest forecasting and sustainable maize production in East Africa.

### 4.1. Climatic Drivers of *C. partellus* Incidence

Temperature was an important climatic variable associated with *C. partellus* incidence across the long‐term farming systems evaluated in this study. Spearman correlations showed a negative relationship between *C. partellus* incidence and minimum, average, and maximum temperatures across all farming systems at Chuka and within the organic farming systems at Thika. However, the direction of the temperature relationship differed among the analytical methods. Spearman correlations represented unadjusted associations, whereas the ARIMAX/SARIMAX and Bayesian coefficients represented conditional relationships after accounting for other variables, temporal structure, and, where applicable, lagged effects. Because minimum, average, and maximum temperature are closely related, average temperature was retained as the sole temperature metric in the final multivariable time‐series models to reduce redundancy among temperature predictors. The temperature results should therefore be interpreted as method‐ and context‐dependent associations rather than as evidence of a single uniform effect of temperature on *C. partellus* incidence.

In contrast, rainfall and relative humidity showed weak or inconsistent associations with pest incidence. These findings are consistent with previous studies identifying temperature as one of the principal environmental factors regulating the development, survival, dispersal, and population dynamics of *C. partellus* and other cereal stem borers [25–27]. Temperature strongly influences insect physiology by regulating metabolic activity, developmental rate, reproduction, and survival. Although warmer conditions generally accelerate insect development up to species‐specific thermal optima, temperatures exceeding these optima may reduce survival, fecundity, and population persistence through physiological stress or increased mortality [28, 29]. The negative associations observed in the present study may therefore reflect temperature‐dependent responses operating within the local environmental conditions; however, the observational nature of the data does not allow a specific thermal mechanism to be established. Similar temperature‐dependent responses have been reported for *C. partellus* across eastern and southern Africa, where climatic warming has been associated with shifts in pest abundance, distribution, and altitudinal range [30].

The relatively weak associations observed for rainfall and relative humidity suggest that these variables had a less direct influence on *C. partellus* incidence than temperature under the conditions of the present study. Although rainfall and humidity are known to influence host plant growth, egg survival, larval establishment, and adult activity, their effects are often indirect and may interact with temperature, crop phenology, and local environmental conditions. Consequently, their individual contributions may be less apparent when evaluated independently over long time series. Similar observations have been reported in other studies of cereal stem borers, where temperature consistently explained a greater proportion of temporal variation than rainfall or humidity [25, 27].

### 4.2. Temporal Lags and Context Specificity

The present study demonstrated that the relationships between climatic variables and *C. partellus* incidence were not uniform across agroecological zones, indicating that pest responses to climatic variability were context dependent. Cross‐correlation analyses revealed contemporaneous associations at lag 0 and short delayed associations at positive lags of 1‐2 quarters. Given the order of the variables in the ccf() analysis, these positive lags indicate that climatic observations preceded the associated changes in *C. partellus* incidence by up to two‐quarters. However, the strength and direction of these associations differed among farming systems and between sites. Similarly, Bayesian analysis indicated that models incorporating current climatic variables provided the best fit for Chuka, whereas models incorporating lagged climatic variables were better supported for Thika. Together, these findings suggest that the influence of climate on *C. partellus* populations varied according to local environmental conditions and farming system context.

The support for lagged climatic effects at Thika may reflect the cumulative influence of antecedent weather conditions on host plant development, pest survival, and seasonal population establishment. In relatively drier agroecosystems such as Thika, rainfall, and temperature during the preceding season may influence crop establishment and host plant availability, thereby affecting the timing and abundance of subsequent *C. partellus* populations. Similar delayed responses of insect pests to antecedent climatic conditions have been reported for cereal stem borers and other agricultural pests in environments where seasonal rainfall strongly regulates crop growth and resource availability [31].

In contrast, the better performance of models incorporating current climatic variables at Chuka suggests that *C. partellus* incidence was more closely associated with prevailing environmental conditions in the analyzed observations. Differences in rainfall regime, humidity, crop conditions, and other local environmental characteristics may contribute to the contrasting temporal relationships observed between the two sites. Comparable site‐specific differences in climatic responses have been reported for insect pests across contrasting agroecological environments [31].

### 4.3. Performance and Limitations of ARIMA‐Based Modeling

The ARIMA models represented the general temporal patterns of *C. partellus* incidence, although measures of model fit varied among farming systems and agroecological zones. Seasonal ARIMA structures provided the best fit for most farming systems at Chuka, whereas relatively simpler ARIMA models were sufficient for several farming systems at Thika. This difference was broadly consistent with the autocorrelation analyses, which indicated generally weak temporal dependence in *C. partellus* incidence, particularly at Thika. The Ljung–Box tests did not provide evidence of substantial residual autocorrelation in the selected models, although these diagnostic results alone do not establish overall model adequacy or out‐of‐sample predictive performance.

Other measures of model fit varied among farming systems. The model for the organic high‐input system at Chuka had an in‐sample *R* ^2^ of 0.650 calculated from the observed and fitted back‐transformed proportions. This value describes how well the selected time‐series model represented the observed temporal variation in *C. partellus* incidence and should not be interpreted as evidence of greater ecological stability or out‐of‐sample forecasting ability. The variation in model fit among sites and farming systems may be associated with differences in climatic conditions, management practices, and other ecological factors affecting pest populations.

The fitted ARIMA models also had limitations. Although they broadly represented the long‐term decline in *C. partellus* incidence, they did not fully capture several incidence peaks during the early part of the monitoring series analyzed. In this dataset, the models represented the general temporal pattern more closely than abrupt increases in incidence. Such differences may arise because pest populations are influenced by multiple interacting biotic and abiotic factors that may not be fully represented in the fitted models. Accordingly, the models should be interpreted primarily as descriptive time‐series models rather than as validated forecasting tools [32].

Future studies could examine whether combining ARIMA‐based models with complementary approaches improves the representation of nonlinear relationships and interactions among climatic and ecological variables. Hybrid approaches that combine ARIMA with machine‐learning techniques have shown potential for analyzing complex agricultural time series [33, 34]. In the present study, ARIMA‐based models provided an interpretable framework for describing temporal patterns in pest incidence. These findings may therefore inform the development and independent validation of future climate‐informed forecasting models.

### 4.4. Farming System Effects After Accounting for Climatic Variability

Bayesian generalized linear mixed modeling demonstrated that farming system effects on *C. partellus* incidence differed according to input level after accounting for climatic covariates and temporal variation. Under high‐input management, *C. partellus* incidence was higher in the organic than in the conventional farming systems at both Chuka and Thika, with the corresponding 95% CrIs excluding zero. In the low‐input systems, the 95% CrIs for farming system effects overlapped zero, providing no clear evidence of differences between organic and conventional systems. These findings indicate that climatic variability alone did not fully explain the observed differences in *C. partellus* incidence and suggest that management‐related factors also contributed to long‐term pest dynamics.

The higher *C. partellus* incidence observed in the organic high‐input systems should be interpreted cautiously. Organic and conventional farming systems differ in several management practices, including nutrient sources, pest management strategies, and soil fertility management, all of which may influence crop growth, host plant characteristics, and pest populations. Consequently, the observed differences may reflect the combined influence of multiple management‐related factors rather than the effect of any single practice. Similar interactions between farming system management and insect pest dynamics have been reported in long‐term agricultural experiments, where pest populations respond to the combined effects of crop management, environmental conditions, and agroecosystem processes [35].

In the low‐input systems, the posterior estimates did not provide clear evidence of differences between organic and conventional farming systems at either site because the corresponding 95% CrIs overlapped zero. One possible explanation is that management contrasts between farming systems were less pronounced under low‐input conditions because both systems received relatively fewer external inputs and were subject to greater environmental limitations. Consequently, climatic variability may have exerted a proportionally greater influence on pest dynamics than management practices under these conditions. Similar interactions between management intensity and agroecosystem performance have been reported in long‐term farming system comparisons [16]. However, further studies incorporating additional ecological variables are needed to test this hypothesis.

Although the present study demonstrates that farming system differences remained evident after accounting for climatic variation, it did not directly evaluate the ecological mechanisms responsible for these differences. Factors such as crop nutritional quality, natural enemy abundance, soil biological activity, and broader agroecosystem processes may also influence *C. partellus* populations and should therefore be considered in future investigations [36]. Furthermore, because the Bayesian models included years but did not explicitly represent repeated observations through a plot‐level random effect, the estimated farming system effects should be interpreted with this model structure into consideration. Overall, the findings suggest that long‐term farming system management is associated with variation in *C. partellus* incidence alongside climatic variability, highlighting the importance of considering both climatic and management factors when developing climate‐informed pest management strategies [16].

### 4.5. Implications for Climate‐Informed Pest Management

The findings of this study suggest that climatic variability, particularly temperature, should be considered when developing pest‐monitoring and forecasting strategies for *C. partellus* in maize production systems. The observed associations between temperature and *C. partellus* incidence varied across sites and farming systems, indicating that climatic relationships may depend on local agroecological conditions. Although *C. partellus* incidence remained below the 20% reference level during much of the available monitoring series, this should not be interpreted as evidence that pest pressure remained below a formally established economic threshold for *C. partellus*. Rather, the 20% level is used here as a descriptive reference for comparing temporal patterns in incidence, as economic or action thresholds may vary with pest species, crop developmental stage, damage metric, and production context [17, 18]. Future forecasting models should therefore consider location‐specific climatic and management factors and undergo independent validation before being used to guide pest management. If adequately validated against appropriate pest‐specific decision thresholds, such models could help inform the timing of integrated pest management interventions.

The Bayesian analyses indicated that differences among farming systems remained under high‐input management after accounting for the climatic variables included in the models. This finding suggests that both climatic conditions and management practices contribute to long‐term *C. partellus* dynamics and should therefore be considered jointly when developing pest management strategies. Rather than relying solely on climatic variables, future forecasting frameworks may benefit from incorporating additional management and ecological information to improve their ability to explain variation in pest populations.

Although the ARIMA models developed in this study were not validated as operational forecasting tools, they provided a description of the observed long‐term temporal patterns in *C. partellus* incidence. These findings may inform the development and evaluation of future climate‐informed pest‐prediction models. Following appropriate validation, such modeling approaches could be integrated with routine weather monitoring, farm advisory services, and decision‐support systems to help guide the timing of field scouting, biological control releases, and other integrated pest management interventions [10, 15].

Future research on climate‐informed pest prediction could explore the integration of time‐series methods with remote sensing, sensor networks, and artificial intelligence–based analytical tools. Such approaches may improve the ability to capture complex ecological interactions and nonlinear pest responses while providing higher spatial and temporal resolution for pest risk assessment [32–34, 37]. However, their usefulness should be evaluated using independent data and they should complement rather than replace long‐term ecological observations such as those used in the present study.

### 4.6. Limitations and Suggestions for Future Work

The present study provides valuable insights into the long‐term relationships between climatic variability and *C. partellus* incidence within contrasting organic and conventional farming systems. However, several limitations should be considered when interpreting the findings.

1. Temporal coverage and aggregation: Although the SysCom long‐term farming system experiment was established in 2007, *C. partellus* monitoring observations were not available continuously for every calendar year represented by the long‐term experiment. The temporal analyses therefore represent the available quarterly monitoring observations rather than uninterrupted surveillance throughout the entire period since the establishment of the experiment. In addition, the original biweekly field observations were aggregated to quarterly observations for the temporal analyses. Consequently, short‐term fluctuations in *C. partellus* populations and some transient outbreak events may not be fully represented by the quarterly series. Higher‐frequency and temporally continuous monitoring would strengthen the characterization of rapid population changes and the independent validation of future forecasting models.
2. Experimental and analytical structure: The field experiment comprised replicated plots and repeated pest assessments, whereas the time‐series analyses were conducted using observations summarized at the farming system and quarterly level. In addition, the Bayesian models included year as a random intercept for interannual variation but did not explicitly represent repeated observations within individual plots through a plot‐level random effect. These aspects of the analytical structure should be considered when interpreting farming system effects and temporal associations.
3. Ecological drivers beyond climate: The analyses focused primarily on climatic variables because these are among the principal environmental drivers of insect population dynamics. However, *C. partellus* populations are also influenced by a range of biological and management‐related factors, including crop phenology, host plant nutritional status, soil moisture, natural enemy abundance, landscape composition, and pest management practices. For example, parasitoids and other natural enemies are known to contribute to the regulation of cereal stem‐borer populations in East Africa [36]. Incorporating these variables into future models could improve understanding of the mechanisms underlying temporal variation in pest incidence.
4. Model assumptions and nonlinear dynamics: ARIMA models provide a useful framework for analyzing linear temporal relationships but may be less effective in representing nonlinear responses, threshold effects, and abrupt ecological changes associated with complex agroecosystems. Although the models provided a description of the observed long‐term patterns in *C. partellus* incidence, they underestimated several outbreak peaks. Additional modeling approaches may therefore improve representation of complex pest dynamics and should be evaluated using independent data before being considered for operational forecasting [32–34].
5. Generalizability of the models: The models developed in this study were based on two long‐term experimental sites representing contrasting agroecological zones in Kenya. While these sites provide a valuable platform for examining climate–pest relationships under well‐characterized management conditions, caution should be exercised when extrapolating the models to production systems or regions with different environmental conditions, cropping practices, or pest management strategies. Independent validation and, where appropriate, site‐specific model calibration would be required before wider application.

Future research should build upon these findings by integrating climatic, biological, and management variables within more comprehensive modeling frameworks. Combining long‐term ecological datasets with complementary analytical approaches, including hybrid time‐series and machine‐learning models, may improve the representation and prediction of *C. partellus* dynamics under changing climatic conditions. In addition, validating forecasting models using independent datasets and evaluating their performance under future climate scenarios would strengthen their potential application in climate‐informed integrated pest management.

## 5. Conclusion

This study indicates that climatic variability and farming system management are both associated with the long‐term variations in *C. partellus* incidence in maize production systems of central Kenya. Across the available long‐term monitoring observations, associations between temperature and *C. partellus* incidence were observed in several farming systems, whereas rainfall and relative humidity showed comparatively weak and inconsistent relationships. The observed climatic associations differed between Chuka and Thika, highlighting the importance of considering local agroecological conditions when investigating climate–pest relationships.

The study further indicated that farming system differences remained evident under high‐input management after accounting for the climatic variables included in the Bayesian models, whereas the corresponding CrIs overlapped zero under low‐input management. These findings suggest that the variation in *C. partellus* incidence may reflect both environmental conditions and farming system management rather than climatic variables alone.

The ARIMA‐based modeling framework described the general temporal patterns in *C. partellus* incidence, although model fit varied among farming systems and several incidence peaks were underestimated. The fitted models should therefore be interpreted as descriptive time‐series models rather than validated forecasting tools. The findings may nevertheless inform the development of future climate‐informed pest‐forecasting models incorporating additional ecological variables and independent validation.

Overall, this study provides a long‐term assessment of climate–pest relationships within contrasting organic and conventional farming systems in sub‐Saharan Africa. The findings contribute to understanding the associations among climatic variability, farming system management, and *C. partellus* incidence and provide information that may support the development of more targeted, climate‐informed integrated pest management strategies for sustainable maize production under changing climatic conditions.

## Author Contributions

C.M.T., E.N.K., and N.A. conceived and designed the study. Methodology was developed by C.M.T., E.N.K., N.A., S.W., and D.S. The investigation was carried out by C.M.T., E.N.K., S.W., N.A., and F.M. Data curation was performed by C.M.T., E.N.K., N.A., S.W., and D.S., while formal analysis was conducted by S.W. and D.S. C.M.T., E.N.K., and S.W. prepared the original draft of the manuscript. T.D., N.M., A.W., S.M., N.A., F.M., E.M., and D.S. contributed to manuscript review and editing. Visualization was conducted by C.M.T., E.N.K., S.W., and D.S. Supervision was provided by C.M.T., E.N.K., and N.A., with project administration managed by C.M.T. and E.N.K. Funding acquisition was undertaken by N.A.

## Funding

The authors gratefully acknowledge the financial support for this research by the following organizations and agencies: the Biovision Foundation for Ecological Development (Grant No: 1040); Coop Sustainability Fund (Grant No: 1040); the Liechtenstein Development Service (LED) (Grant No: 1040); and the Swiss Agency for Development and Cooperation (SDC) (Grant No: 1040); additional institutional support was provided by the Swedish International Development Cooperation Agency (Sida), the Australian Centre for International Agricultural Research (ACIAR), the Government of Norway, the German Federal Ministry for Economic Cooperation and Development (BMZ), and the Government of the Republic of Kenya.

## Disclosure

All authors read and approved the final manuscript. The views expressed in this publication do not necessarily reflect those of the funding agencies.

## Conflicts of Interest

The authors declare no conflicts of interest.

## Supporting Information

Additional supporting information can be found online in the Supporting Information section.

## Supporting information


**Supporting Information** Supporting Table S1: Posterior estimate for the Bayesian mixed‐effects model fitted to *Chilo partellus* incidence at Chuka. Supporting Table S2: Posterior estimate for the Bayesian mixed‐effects model fitted to *Chilo partellus* incidence at Thika.

## Data Availability

The data files supporting the findings of this study will be made available without undue reservation upon reasonable request.
