# Supplementary material for: Long‐Term Population Dynamics of the Spotted Stem Borer (Chilo partellus) in Contrasting Maize Farming Systems Under Variable Climatic Conditions in East Africa
Source: Scientifica (Cairo). 2026 Oct 21;2026:7137270. doi: 10.1155/sci5/7137270 (PMC13686372; doi:10.1155/sci5/7137270)
Supplement: Supplementary file 1 [file SCI5-2026-7137270-s001.zip › Suppl Tables_Table S1 and S2_Clean.docx]

**Supplementary Tables (S1 and S2)**

Supplementary Table S1: Posterior estimate for the Bayesian mixed-effects model fitted to *Chilo partellus* incidence at Chuka.

|  | High input system - Chuka | | |  |
| --- | --- | --- | --- | --- |
| Parameter | Estimate | s.e | Lower 95% Cr.I | Upper 95% Cr.I |
| Intercept | -5.61 | 0.93 | -7.47 | -3.7 |
| **Treatment: Org_High** | **0.34** | **0.09** | **0.15** | **0.52** |
| Average Temperature | 0.04 | 0.04 | -0.05 | 0.13 |
| Average RH | 0.01 | 0.01 | -0.02 | 0.04 |
| Rainfall | 0 | 0.01 | -0.02 | 0.02 |
| Quarter2 | 0.48 | 0.21 | 0.06 | 0.9 |
| Quarter3 | 1.19 | 0.21 | 0.78 | 1.6 |
| Quarter4 | 1.3 | 0.25 | 0.82 | 1.79 |
| Year variance | 2.34 | 0.67 | 1.38 | 3.95 |
|  | Low input system - Chuka | | |  |
| Intercept | -8.91 | 4.55 | -17.87 | -0.06 |
| **Treatment: Org_Low** | **-0.06** | **0.09** | **-0.25** | **0.12** |
| Average Temperature | 0.09 | 0.21 | -0.32 | 0.5 |
| Average RH | 0.02 | 0.01 | 0 | 0.05 |
| Rainfall | 0.18 | 0.02 | 0.13 | 0.22 |
| Quarter2 | -0.11 | 0.29 | -0.68 | 0.47 |
| Quarter3 | 2.97 | 0.43 | 2.12 | 3.83 |
| Quarter4 | 0.51 | 0.28 | -0.05 | 1.06 |
| Year variance | 2.53 | 0.73 | 1.47 | 4.29 |

s.e – standard error. For high input system, Treatment: Conv_High was set as reference and for low input system Treatment: Conv_Low was set as reference, for factor Quarter, Quarter1 was set as reference, Cr.l. is Credible interval.

Supplementary Table S2: Posterior estimate for the Bayesian mixed-effects model fitted to *Chilo partellus* incidence at Thika.

|  | High input system - Thika | | |  |
| --- | --- | --- | --- | --- |
| Parameter | Estimate | s.e | Lower 95% Cr.I | Upper 95% Cr.I |
| Intercept | -3.90 | 1.10 | -5.98 | -1.59 |
| Treatment: Org_High | 1.02 | 0.15 | 0.72 | 1.33 |
| Lagged Temperature | 1.02 | 0.12 | 0.78 | 1.25 |
| Lagged RH | -0.08 | 0.07 | -0.23 | 0.06 |
| Lagged Rainfall | -1.26 | 0.23 | -1.70 | -0.82 |
| Quarter2 | -1.39 | 0.22 | -1.83 | -0.97 |
| Quarter3 | 0.68 | 0.32 | 0.07 | 1.32 |
| Quarter4 | -1.98 | 0.39 | -2.76 | -1.23 |
| Year variance | 2.72 | 0.74 | 1.63 | 4.53 |
|  | Low input system - Thika | | |  |
| Intercept | -3.33 | 0.60 | -4.51 | -2.08 |
| Treatment: Org_Low | 0.05 | 0.12 | -0.20 | 0.29 |
| Lagged Temperature | -0.06 | 0.13 | -0.31 | 0.20 |
| Lagged RH | -0.58 | 0.08 | -0.74 | -0.43 |
| Lagged Rainfall | 0.05 | 0.16 | -0.27 | 0.36 |
| Quarter2 | -0.55 | 0.21 | -0.96 | -0.13 |
| Quarter3 | 0.39 | 0.25 | -0.09 | 0.88 |
| Quarter4 | -0.26 | 0.33 | -0.91 | 0.39 |
| Year variance | 1.48 | 0.48 | 0.85 | 2.66 |

s.e – standard error. For high input system, Treatment: Conv_High was set as reference and for low input system Treatment: Conv_Low was set as reference, for factor Quarter, Quarter1 was set as reference. Cr.l. is Credible interval.
